# Potential of conservation agriculture modules for energy conservation and sustainability of rice-based production systems of Indo-Gangetic Plain region

**DOI:** 10.1007/s11356-020-10395-x

**Published:** 2020-08-18

**Authors:** Rajiv Nandan, Shish Pal Poonia, Sati Shankar Singh, Chaitanya Prasad Nath, Virender Kumar, Ram Kanwar Malik, Andrew McDonald, Kali Krishna Hazra

**Affiliations:** 1 Sam Higginbotom Institute of Agriculture Technology and Sciences, Allahabad, Uttar Pradesh 211007 India; 2 International Maize and Wheat Improvement Center (CIMMYT)—India, NASC Complex, DPS Marg, New Delhi, 110012 India; 3 ICAR–Agricultural Technology Application Research Institute, Bhumi Vihar Complex, Sector-III, Salt Lake, Kolkata, West Bengal 700097 India; 4 Crop Production Division, ICAR–Indian Institute of Pulses Research (ICAR–IIPR), Kanpur, Uttar Pradesh 208024 India; 5 International Rice Research Institute, DAPO 7777, Metro Manila, Philippines; 6 Soil and Crop Sciences Section, School of Integrative Plant Science, Cornell University, Ithaca, NY USA; 7 Agriculture and Food engineering Department, Indian Institute of Technology Kharagpur, Kharagpur, West Bengal 721302 India

**Keywords:** Conservation agriculture, Crop residue retention, Crop establishment, Direct seeded rice, Energy budgeting, Rice/maize/wheat system

## Abstract

Rice-based cropping systems are the most energy-intensive production systems in South Asia. Sustainability of the rice-based cropping systems is nowadays questioned with declining natural resource base, soil degradation, environmental pollution, and declining factor productivity. As a consequence, the search for energy and resource conservation agro-techniques is increasing for sustainable and cleaner production. Conservation agriculture (CA) practices have been recommended for resource conservation, soil health restoration and sustaining crop productivity. The present study aimed to assess the different CA modules in rice-based cropping systems for energy conservation, energy productivity, and to define energy-economic relations. A field experiment consisted of four different tillage-based crop establishment practices (puddled-transplanted rice followed by (*fb*) conventional-till maize/wheat (CTTPR-CT), non-puddled transplanted rice *fb* zero-till maize/wheat (NPTPR-ZT), zero-till transplanted rice *fb* zero-till maize/wheat (ZTTPR-ZT), zero-till direct-seeded rice *fb* zero-till maize/wheat (ZTDSR-ZT)), with two residue management treatments (residue removal, residue retention) in rice–wheat and rice–maize rotations were evaluated for energy budgeting and energy-economic relations. Conservation-tillage treatments (NPTPR-ZT, ZTTPR-ZT, and ZTDSR-ZT) reduced the energy requirements over conventional tillage treatments, with the greater reduction in ZTTPR-ZT and ZTDSR-ZT treatments. Savings of energy in conservation-tillage treatments were attributed to reduced energy use in land preparation (69–100%) and irrigation (23–27%), which consumed a large amount of fuel energy. Conservation-tillage treatments increased grain and straw/stover yields of crops, eventually increased the output energy (6–16%), net energy (14–26%), energy ratio (25–33%), and energy productivity (23–34%) as compared with CTTPR-CT. For these energy parameters, the treatment order was ZTDSR-ZT ≥ ZTTPR-ZT > NPTPR-ZT > CTTPR-CT (*p* < 0.05). Crop residue retention reduced net energy, energy ratio, and energy productivity when compared with residue removal. Our results of energy-economic relations favored the “conservative hypothesis,” which envisages that energy and monetary investments are not essentially the determinants of crop productivity. Thus, zero tillage-based crop establishments (ZTTPR-ZT, ZTDSR-ZT) in rice-based production systems could be the sustainable alternative to conventional tillage-based agriculture (CTTPR-CT) as they conserved non-renewable energy sources, reduced water requirement, and increased crop productivity.

**Electronic supplementary material:**

The online version of this article (10.1007/s11356-020-10395-x) contains supplementary material, which is available to authorized users.

## Introduction

The agriculture sector of developing countries has witnessed spectacular progress in farm mechanization that markedly increased the energy inflows in agriculture (Saad et al. 2016; Choudhary et al. 2017). The scale of energy investment and accessible resource base primarily determine the crop productivity and production economics (Shahbaz et al. 2017). Nevertheless, energy and input-intensive production systems have several sustainability concerns (Kumar et al. 2020). Conservation of non-renewable energy sources and efficient resource management in agriculture is increasingly being realized for cleaner and sustainable production (Kumar et al. 2019). So, there is an increased need for developing alternative agro-technique(s) that can substantially reduce the energy requirements in crop production (Saad et al. 2016). The dominated energy concept, i.e., increased energy investments cause higher crop productivity and economic growth (Ouedraogo 2013; Aslan et al. 2014), is contrasted by the “conservative hypothesis” (Narayan 2016; Kasman and Duman 2015). The energy use in crop production, economics, and the environment in a given agro-ecosystem are strongly interrelated, and thus a holistic approach must be adopted to address the evident challenges of energy-intensive production systems (Pimentel et al. 1994).

The increasing scarcity of human labor has increased the pressure for the adoption of the machine-driven operations like tillage, sowing/transplanting, harvesting, and threshing (Jat et al. 2013). The adverse impact of mechanization and input-intensive agricultural practices on soil quality and environmental pollution are becoming the major current concerns (Parihar et al. 2018), demonstrating the need for developing alternative crop management strategies that could minimize the energy use, protect the environment, and maintain comparable or even higher crop productivity over current practices. For such strategic change in production techniques targeting to elevate energy productivity, a detailed input–output energy budgeting is the prerequisite (Tuti et al. 2012).

Rice-based cropping systems are predominant in South Asia (Hazra et al. 2018). The rice–wheat cropping system is extensively being practiced in the Indo-Gangetic Plain (IGP) region (~ 11.7 m ha) and contributes a major share of the national food-grain production (Chauhan et al. 2012; Nandan et al. 2018a). However, conventional rice-based cropping systems are mostly input and energy intensive (Hazra et al. 2019). Land preparation/tillage, wet-tillage (puddling), high rate of fertilizers, and frequent irrigation to maintain standing water during the rice crop season consume a large amount of the energy sources (Nandan et al. 2018b; Lal et al. 2019). Meanwhile, the sustainability of the rice-based cropping systems are primarily threatened by depletion of groundwater level, deterioration of soil health and soil native fertility, declining factor productivity, and environmental pollution due to intensive tillage operations and inappropriate agronomic practices (Nath et al. 2019).

Conservation agriculture (CA), which is nowadays gaining a larger interest in South Asia, offers strategic options to upscale the resource and energy productivity (Kumar et al. 2019a). The benefits of CA over conventional agriculture on soil health (Gathala et al. 2015; Devkota et al. 2019), resource conservation (Nandan et al. 2018b), and ecosystem services (Alam et al. 2019) have already been reported from the tropical IGP regions. However, very limited reports are available on the impact of recently developed CA modules in the rice–wheat or rice–maize cropping systems on energy inflow–outflow balance. The recent developments of conservation tillage cum crop establishment practices in the lowland rice ecologies like non-puddled transplanting of rice (NPTPR), zero-tillage transplanting (ZTTPR), and zero-tillage direct seeding of rice (ZTDSR) fits in the rice-based cropping systems in CA mode. In the present study, the energy budgeting and energy-economic relationship was evaluated in two rice-based cropping systems (rice–wheat and rice–maize) of the IGP region that consisted of different tillage-based crop establishment methods and residue management treatments. The objectives of the study were (1) to assess the scale of energy conservation of different CA modules over conventional practices in rice-based cropping systems, (2) to estimate operation-wise and source-wise energy inflow and energy productivity under different CA modules in rice-based systems, and (3) to derive the association between different energy parameters and production economics variables to define the energy-economic relations.

## Materials and methods

### Site and soil characteristics

**Fig. 1:**
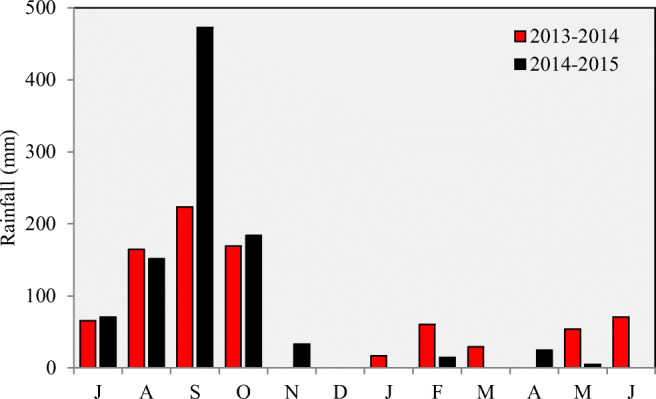
Monthly rainfall (mm) received during the experimental year 2013–2014 and 2014–2015

The field experiment was initiated in the year 2009 at the research farm of Indian council of Agricultural Research- Research Complex for Eastern Region (ICAR–RCER), Patna, Bihar (25°37′ N, 85°13′ E and 36 m above sea level). The climate of the region is subtropical–humid. The soil is clay-loam in texture and comes under the taxonomical class Fluvisol (World Reference Base soil classification). The site receives 1130 mm of annual rainfall, and 85–90% of the rainfall occurs during June to September. The month-wise rainfall during the study period (2013–2015) is presented in Fig. 1.

### Treatment details and experimental design

Treatments comprised two crop rotations (rice (*Oryza sativa* L)–wheat (*Triticum aestivum* L.) and rice–maize (*Zea mays* L.)), two residue management treatments (residue removal and residue retention (~33%)), and four crop establishment/tillage treatments (puddled transplanting of rice followed by conventional tillage in wheat/maize (CTTPR-CT), non-puddled transplanting of rice followed by zero tillage (ZT) in wheat/maize (NPTPR-ZT), zero tillage transplanting of rice followed by zero tillage in wheat/maize (ZTTPR-ZT), and zero tillage direct seeded rice followed by zero tillage in wheat/maize (ZTDSR-ZT)). In CTTPR-CT treatment, the field was prepared with two plowing, two harrowing, one wet-tillage (puddling), and planking, and 21-day-old rice seedlings (2 seedlings hill^−1^) were manually transplanted (20 cm × 15 cm); for winter crops (wheat/maize), the field was conventionally tilled (two plowing, two harrowing, planking) and wheat crop was established by broadcasting, where maize was manually dibbled. In NPTPR-ZT treatment, the field was prepared by two plowing, two harrowing, and planking (no wet-tillage); rice transplanting was done in the same way as CTTPR-CT; wheat and maize crops were sown in zero-tillage condition using zero-till happy-seeder machine. In ZTTPR-ZT plots, no tillage operation was performed, and a day before rice transplanting, the plots were flooded to make the soil soft and rice was transplanted in the same way as CTTPR-CT; wheat and maize crops were sown in zero-tillage condition using zero-till happy-seeder machine. In ZTDSR-ZT treatment, direct seeding of rice was done using zero-till seed cum fertilizer drill in zero-till flat plots at 20-cm row spacing; wheat and maize crops were sown in zero-tillage condition using zero-till happy-seeder machine. In residue retention treatment, the rice and wheat crops were harvested at a height of ~ 30 cm by the combine harvester and maize was harvested at a height of ~ 70 cm to retain approximately 33% of crop residue in the field, whereas in residue removal plots, all the crops were harvested from the ground. The treatments were laid out in split-split-plot design, accommodating crop rotation, residue management, and crop establishment treatments in the main plot, subplot and sub-subplot, respectively, and the treatments were replicated thrice. The dimension of each sub-subplot was 10.5 × 7.5 m.

### Crop management

In ZTDSR-ZT treatment, rice (hybrid ‘Arize Tez’) was directly sown in the main field in zero-till condition using zero-till multi-crop planter. On the same day, rice nursery (bed size 12 × 6 m) was raised. The nursery area was plowed and puddled before sowing. Rice seeds were soaked in water and placed in a gunny bag overnight. The pre-germinated seeds were then uniformly broadcasted and watered at regular intervals. The nursery bed was submerged with a shallow layer of water after a week of sowing. In CTTPR-CT, NPTPR-ZT, and ZTTPR-ZT treatments, 21-day old seedlings were transplanted manually by human labor. A plant spacing of 20 × 15 cm was maintained in all the transplanted rice treatments. For rice crop, the fertilizer N/P_2_O_5_/K_2_O was applied at 120:40:20 kg ha^−1^. Half dose of nitrogen (N) and the full dose of phosphorus (P) and potassium (K) along with 25 kg ha^−1^ zinc sulfate (ZnSO_4_) and 20 kg ha^−1^ sulfur were applied as a basal dose. The remaining half dose of N was applied in two equal splits at active tillering and panicle initiation stages. In ZTDSR-ZT treatment, 18% of N and the full dose of P and K along with 25 kg ha^−1^ zinc sulfate (ZnSO_4_) and 20 kg ha^−1^ sulfur were applied basal and the remaining N (82%) was top-dressed in three equal splits at 15 days after sowing (DAS), active tillering, and panicle initiation stage. Irrigation was applied to rice crop based on the crop requirements for different tillage regimes and rainfall received during the crop season (Fig. 1). For weed control in rice crop, pretilachlor (50% EC) 0.4 kg a.i. ha^−1^ was applied in CTTPR-CT and NPTPR-ZT treatments within 24 h of transplanting without applying any post-emergence herbicides. Pretilachlor was applied at saturated soil condition after draining the water after transplanting. Within 24 h of herbicide application, plots were filled with water to achieve better efficacy of herbicide, whereas in ZTTPR-ZT and ZTDSR-ZT treatments, pre-emergence application of pendimethalin (30% EC) 0.75 kg a.i. ha^−1^ within 2 days of sowing/transplanting followed by post-emergence application of bispyribac sodium (10% SC) at 20 g a.i. ha^−1^ at 25 days of sowing/transplanting were undertaken. For insect pest management in rice, two sprays of imidacloprid was performed.

Wheat (cv*.* HD 2967) and maize (cv*.* ‘Decalb 9120’) were sown during the second fortnight of November. The wheat crop was sown by manually broadcasting in CTTPR-CT treatment, and in the zero-tillage treatments (NPTPR-ZT, ZTTPR-ZT, and ZTDSR-ZT), the crop was sown by zero-till happy-seeder machine maintaining an inter-row spacing of 22.5 cm. Likewise, in CTTPR-CT treatment, maize was sown by manual dibbling method, and in zero-tillage treatments, the crop was sown by zero-till happy-seeder machine with a plant spacing of 60 × 15 cm. Both wheat and maize seeds were treated with systemic insecticide imidacloprid at 7 g kg^−1^ seeds. The fertilizers dose of N/P_2_O_5_/K_2_O at 120:60:40 kg ha^−1^ were applied to the wheat crop. Half of the total amount of N and full doses of P and K were applied at the time of sowing. The remaining dose of N was applied in the form of urea in two equal splits after first (21 DAS) and second (50 DAS) irrigation. Likewise, 150:75:50 kg ha^−1^ of N/P_2_O_5_/K_2_O was supplied to maize crop; half quantity of N and full doses of P and K were applied at the time of sowing. The remaining dose of N was applied in two equal split doses after 60 DAS and taselling time. In addition to the N, P, and K fertilizers, 25 kg ha^−1^ zinc sulfate (ZnSO_4_) and 20 kg ha^−1^ sulfur were applied as basal dose to both wheat and maize crops. Before the sowing of wheat and maize crop, pre-plant application of glyphosate (41% SL) 1.5 kg a.i. ha^−1^ was applied in NPTPR-ZT, ZTTPR-ZT, and ZTDSR-ZT treatments. After that, post-emergence application of ready mix herbicide sulfosulfuron (75% WG) + metsulfuron (5% WG) at 32 g a.i. ha^−1^ was applied to wheat crop irrespective of tillage treatments at 25 DAS. In maize crop, post-emergence application of atrazine (50% WP) 1.25 kg ha^−1^ at 25 DAS was applied irrespective of treatments. Herbicides were applied with knapsack sprayer fitted with flat-fat nozzle with 400 l ha^−1^ water. A total of six irrigations were applied to wheat crop (crown root initiation, active tillering, booting, flowering, dough stages), where, in maize crop, a total of five irrigations were applied at different crop growth stages (20–25 days interval). The total depth of irrigation applied to the rice, wheat, and maize crop in different treatment is given in Table 1.

**Table 1:** Seed rate, tillage type and frequency, fertilizer N splits, total irrigation depth, and weed control measures for the component crops under different tillage cum crop establishment treatments

Treatment		Seed rate (kg ha^−1^)	Tillage (*n*)				N split (*n*)	Irrigation depth (cm)^$^	Hand weeding (*n*)	Pre-emergence/sowing herbicide	Post-emergence herbicide
			PT	H	P	WT					
CTTPR-CT	Rice	15	2	2	1	1	3	390	–	Pretilachlor	–
	Wheat	120	2	2	1	–	3	90	–	–	Sulfosulfuron + metsulfuron
	Maize	20	2	2	1	–	3	75	–	–	Atrazine
NPTPR-ZT	Rice	15	2	2	1	–	3	260	–	Pretilachlor	–
	Wheat	100	–	–	–	–	3	90	–	Glyphosate	Sulfosulfuron + metsulfuron^#^
	Maize	20	–	–	–	–	3	75	–	Glyphosate	Atrazine
ZTTPR-ZT	Rice	15	–	–	–	–	3	260	–	Pendimethalin	Bispyribac Na
	Wheat	100	–	–	–	–	3	90	–	Glyphosate	Sulfosulfuron + metsulfuron
	Maize	20	–	–	–	–	3	75	–	Glyphosate	Atrazine
ZTDSR-ZT	Rice	25	–	–	–	–	4	280	1–2*	Pendimethalin	Bispyribac Na
	Wheat	100	–	–	–	–	3	90	–	Glyphosate	Sulfosulfuron + metsulfuron
	Maize	20	–	–	–	–	3	75	–	Glyphosate	Atrazine

*PT* preparatory tillage, *H* harrowing, *P* plowing, *WT* wet-tillage (puddling)

^$^Average value of 2 years (2013–2014 and 2014–2015)

*Hand weeding was done in ZTDSR-ZT due to higher weed growth

#Ready-mix herbicide 75% sulfosulfuron + 5% WG metsulfuron

#### Grain and straw yield estimation

To estimate the grain and straw/stover yields of component crops, a net plot area of 3 × 3 m was manually harvested, threshed, and weighed. Then, a subsample of the harvested grain was used for estimation of moisture content. The grain yield of all the component crops was adjusted at 14% moisture content (*w*/*w*).

### Energy calculation and budgeting

The study aimed to compare the conventional and CA-based practices on energy parameters in the fourth and fifth year of crop rotations, i.e., year 2013–2014 and 2014–2015. The energy input–output relationship in different crop production systems was derived and energy inflow–outflow budgeting was done. The different sources of energy in crop production were computed based on the input requirement and their corresponding energy coefficient given in Table 2. According to Devasenapathy et al., the energy sources are primarily classified into two categories, namely, direct and indirect energy sources (Devasenapathy et al. 2009). In the present study, direct energy sources including diesel, tractors, and stationary motors animate power (human and animal). Besides this, rain, wind, solar radiation, and so on are also listed under direct energy sources, but in the present study, these energy sources are not taken into account. On the other hand, indirect energy sources are those which do not release energy directly but dissipate energy during various conversion processes (Saad et al. 2016). The energy required in manufacturing, storage, and transportation activities contributes to the indirect energy calculation. For the present study, seeds, crop residues, fertilizers, chemicals, and machinery are categorized under the sources of indirect energy. Following the guidelines of earlier studies, the nutrient removal by crop(s) and energy involved in the changes in soil organic carbon was not considered in the present study.

**Table 2:** Energy coefficient of different energy sources used in the study

Particular			Unit	Energy coefficient (MJ unit^−1^)	Reference
Input	Prime movers	(tractor, 5-hp motor)	kg	64.8	Devasenapathy et al. (2009)
	Farm machinery	(disc harrow, cultivator, seed drill, dehusker-cum-sheller, sprayer)	kg	62.7	Devasenapathy et al. (2009)
	Combine harvester		kg	83.5	Devasenapathy et al. (2008)
	Diesel including lubricant		l	56.31	Devasenapathy et al. (2008, 2009); Chaudhary et al. (2009)
	Irrigation water		m^3^	1.02	Azarpour (2012)
	Human power	Adult man	Man-hour	1.96	Devasenapathy et al. (2008, 2009); Chaudhary et al. (2009)
		Adult woman	Woman-hour	1.57	Devasenapathy et al. (2008, 2009)
	Chemical fertilizer	N fertilizer	kg	60.6	Devasenapathy et al. (2008, 2009); Tuti et al. (2012)
		P_2_O_5_ fertilizer	kg	11.1	Devasenapathy et al. (2008, 2009); Tuti et al. (2012)
		K_2_O fertilizer	kg	6.7	Devasenapathy et al. (2008, 2009); Tuti et al. (2012)
	Superior chemical	Granular	kg	120	Devasenapathy et al. (2008, 2009); Chaudhary et al. (2009)
		Liquid	ml	0.102	Chaudhary et al. (2009); Devasenapathy et al. (2009)
Output	Main product	Rice grain	kg	15.1	Devasenapathy et al. (2008)
		Maize grain	kg	15.7	Devasenapathy et al. (2008)
		Wheat grain	kg	15.1	Devasenapathy et al. (2008)
	By-product	Straw/stover	kg	12.5	Devasenapathy et al. (2008)

All the energy sources were converted to energy unit of megajoule (MJ). The primary data on various inputs and agronomic operations during the cropping years 2013–2014 and 2014–2015 were used for estimation of energy calculation. Energy coefficients are used as the standard conversion factors for calculation of energy content in a compound or potential to perform a work by different sources. For calculation of energy investment in the form of man-day or woman-day hour, their values were multiplied by the energy coefficient 1.96 and 1.57 MJ per hour, respectively. As a standard assessment, 1 man-day is equivalent to 0.8 woman-day. Energy coefficient in grains or crop biomass is the total calorific value of carbohydrate, protein, and fat content per unit mass. The energy coefficients (Table 2) from various available literature of each item were adopted (Devasenapathy et al. 2009; Tuti et al. 2012; Saad et al. 2016; Choudhary et al. 2017) to estimate input and output energy (expressed as MJ ha^−1^).

Energy equivalents of all inputs were summed to get an estimate for the total input energy. Energy utilization in farm operations was calculated based on energy consumed in land preparation, sowing or transplanting, fertilizer management, irrigation, intercultural operation, plant protection, harvesting, and threshing. The source-wise renewable and non-renewable energy under direct and indirect energies of inputs were also calculated, namely, human labor, water, seed, crop residue, diesel, agrochemicals (pesticides and herbicides), fertilizers, and machinery. The grain and straw/stover yields of rice, maize, and wheat crops and their equivalent yields were converted in terms of energy (MJ ha^−1^) using corresponding energy coefficients given in Table 2.

### Calculation of energy indices

Output energy, defined as the sum grain and straw/stover energy equivalents, was calculated by the following formula

1 $$ \mathrm{Output}\ \mathrm{energy}\ \left(\mathrm{MJ}\ {\mathrm{ha}}^{-1}\right)=\left[\mathrm{grain}\ \mathrm{yield}\ \left(\mathrm{kg}\ {\mathrm{ha}}^{-1}\right)\times \mathrm{energy}\ \mathrm{coefficient}\ \mathrm{of}\ \mathrm{grain}\ \left(\mathrm{MJ}\ {\mathrm{kg}}^{-1}\right)\left]+\right[\mathrm{straw}\ \mathrm{yield}\ \left(\mathrm{kg}\ {\mathrm{ha}}^{-1}\right)\times \mathrm{energy}\ \mathrm{coefficient}\ \mathrm{of}\ \mathrm{straw}\ \left(\mathrm{MJ}\ {\mathrm{kg}}^{-1}\right)\right] $$

An accounting approach is used to analyze some basic measures of input–output energy relation like net energy return, energy ratio, and energy productivity (Devasenapathy et al. 2009; Tuti et al. 2012; Choudhary et al. 2017; Kumar et al. 2019b). Net energy return, defined as the difference between the total output energy produced and total input energy required, was calculated using the following formula:

2 $$ \mathrm{Net}\ \mathrm{energy}\ \mathrm{return}\kern0.5em \left(\mathrm{MJ}\ {\mathrm{ha}}^{-1}\right)=\left[\mathrm{output}\ \mathrm{energy}\ \left(\mathrm{MJ}\ {\mathrm{ha}}^{-1}\right)-\mathrm{input}\ \mathrm{energy}\left(\mathrm{MJ}\ {\mathrm{ha}}^{-1}\right)\right] $$

3 $$ \mathrm{Energy}\ \mathrm{ratio}=\frac{\mathrm{Output}\ \mathrm{energy}\ \left(\mathrm{MJ}\ {\mathrm{ha}}^{-1}\right)}{\mathrm{Input}\ \mathrm{energy}\ \left(\mathrm{MJ}\ {\mathrm{ha}}^{-1}\right)} $$

4 $$ \mathrm{Energy}\ \mathrm{productivity}\kern0.5em \left(\mathrm{kg}\ {\mathrm{MJ}}^{-1}\right)=\frac{\mathrm{Crop}\ \mathrm{or}\ \mathrm{system}\ \mathrm{yield}\ \left(\mathrm{kg}\ {\mathrm{ha}}^{-1}\right)}{\mathrm{Input}\ \mathrm{energy}\ \left(\mathrm{MJ}\ {\mathrm{ha}}^{-1}\right)} $$

### Economic analysis

The economic analysis of each treatment was calculated based on the prevailing market price of all the inputs and outputs. For the present study, the variable cost of cultivation includes tillage operations, seed rate, machinery, transplanting/sowing operations, human labor, plant protection chemicals, irrigation, harvesting, and threshing. All the costs (both fixed and variable cost) were then summed up to estimate the cost of cultivation, and this is expressed in Indian national rupee per hectare basis (INR ha^−1^). The grain yields of the component crops were converted to monetary value using the minimum support price (MSP), Government of India for the year. Likewise, the economic return from the straw/stover outputs was calculated based on the regional market price and quantity of straw/stover outputs under different treatments. Then, the total monetary return from grain and straw was summed up to estimate the gross return. The net returns were calculated as the difference between gross returns and total variable cost. The benefit–cost ratio (BCR) was estimated by dividing net returns with total variable cost.

### Statistical analysis

Data were subjected to analysis of variance (ANOVA) of split-split-plot design using online statistical program OPSTAT (Sheoran et al. 1998). Statistical analysis was performed for the parameters namely output energy, net energy, and energy ratio and energy productivity only. However, for input energy components, no statistical analysis was done as these parameters did not vary within replications. The least significant difference (LSD) was calculated at *α* = 0.05 and used for comparison of treatments means. Principal component analysis (PCA) was done in Window-based software PAST 3.14. Heat map presentation with cluster analysis was done using Heatmapper: web-enabled heat mapping tool (Babicki et al. 2016).

## Results

### Energy inputs

**Fig. 2:**
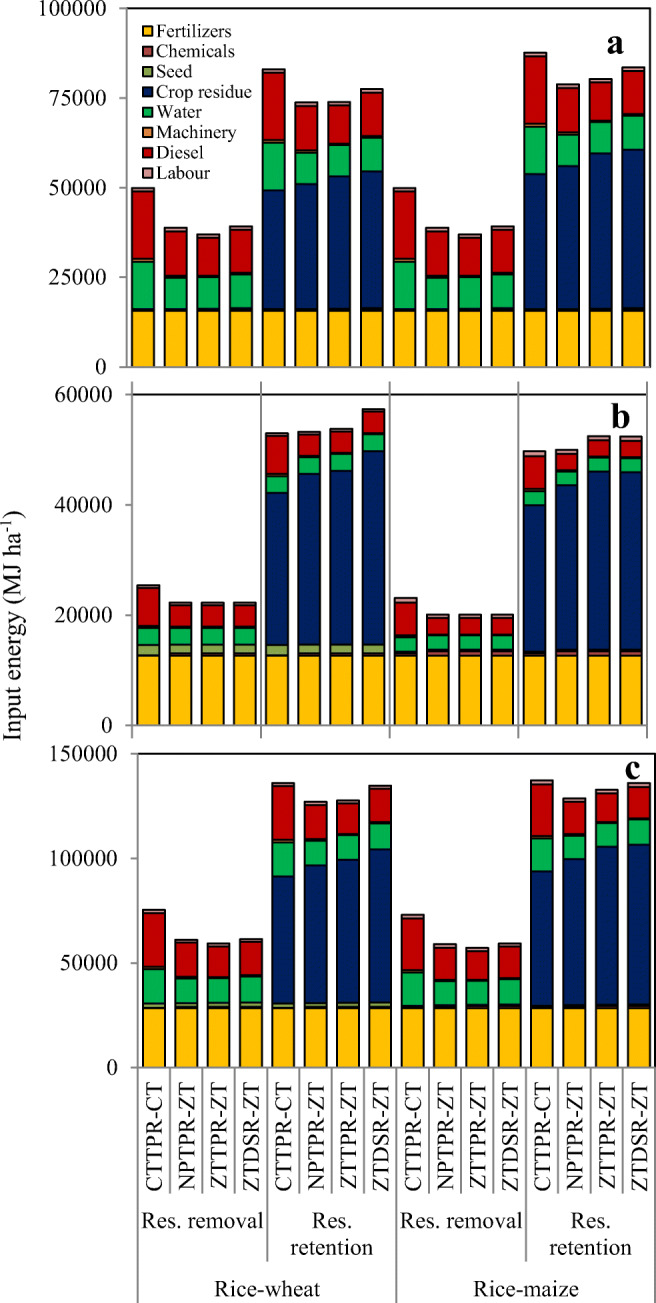
Source-wise input energy (MJ ha^−1^) components in rainy season rice crop (**a**), winter season wheat/maize crop (**b**), and rice–maize/wheat system (**c**) under different treatments. The values are the mean of 2 years (2013–2014 and 2014–2015)

The total energy investment in rice cultivation was markedly higher than wheat and maize cultivation (Fig. 2). The total energy use in rice crop was 38 and 36% higher over wheat and maize crops. Among the different sources of energy, diesel, fertilizers, irrigation, and crop residue (in residue retention treatments) together accounted for 96% of total energy input in rice cultivation. Crop residue was the primary bio-energy input component in residue retention treatments. On average, the residue retention increased the energy use (75% higher) over residue removal treatment (Table 3). Conservation tillage treatments (NPTPR-ZT, ZTTPR-ZT, and ZTDSR-ZT) reduced the energy input for irrigation (29–34%), diesel (34–43%), and machinery (31–52%) compared with conventional tillage practice (CTTPR-CT) (Fig. 2). Subsequently, the saving of total input energy in NPTPR-ZT, ZTTPR-ZT, and ZTDSR-ZT treatments was 16, 18, and 13%, respectively, as compared with CTTPR-CT treatment (Table 3). Likewise, the operation-wise energy use pattern revealed that in rice crop, higher energy requiring operations were crop residue management followed by irrigation (Table 3). Conservation tillage treatments reduced energy use in land preparation (1264–3272 MJ ha^−1^), irrigation (8314–9843 MJ ha^−1^), and increased the energy input through crop residue (1029–2896 MJ ha^−1^) as compared with conventional tillage-based agriculture (CTTPR-CT).

**Table 3:** Operation-wise energy use (MJ ha^−1^) in rice crop as influenced by cropping system, residue management, and TCE practices (2-year mean)

Treatment	Land preparation	Residue management	Sowing/transplanting	Fertilizer management	Irrigation	Inter-culture	Chemical weed management	Plant protection	Harvest/threshing	Total input energy
	(MJ ha^−1^)									
Cropping system										
Rice–wheat	1320	17,878	653	15,764	22,723	53	243	68	450	59,152
Rice–maize	1320	20,638	653	15,764	22,723	53	243	68	450	61,913
Residue management										
Residue removal	1320	–	653	15,764	22,723	35	243	68	450	41,257
Residue retention	1320	38,516	653	15,764	22,723	71	243	68	450	79,808
TCE practice										
CTTPR-CT	3272	17,690	518	15,764	29,723	–	155	68	450	67,641
NPTPR-ZT	2008	18,719	518	15,764	19,880	–	155	68	450	57,563
ZTTPR-ZT	–	20,037	518	15,764	19,880	–	331	68	450	57,049
ZTDSR-ZT	–	20,586	1056	15,764	21,409	212	331	68	450	59,876

In wheat, fertilizers, irrigation, diesel, and crop residue (in residue retention treatments) were the primary components of total energy input. Conservation tillage treatments (NPTPR-ZT, ZTTPR-ZT, and ZTDSR-ZT) reduced the energy requirement in machinery (227 MJ ha^−1^), diesel (2957 MJ ha^−1^), and labor (45 MJ ha^−1^). Subsequently, conservation tillage-based treatments reduced the total energy requirement by 3186 MJ ha^−1^ in residue removal treatments. However, conservation tillage treatments increased the energy input in the residue retention treatments (Table 4) due to higher crop residue inputs. In maize, conservation tillage practices reduced the total energy requirement by 3051 MJ ha^−1^, which was primarily because of curtailing the use of diesel energy (2957 MJ ha^−1^). Energy input in land preparation (3272 MJ ha^−1^) was completely saved in conservation tillage-based crop establishment practices. The energy input through crop residue constitutes about 39 and 45% in wheat and maize cropping (Table 4).

**Table 4:** Operation-wise energy use (MJ ha^−1^) in wheat and maize crop as influenced by cropping system, residue management, and TCE practices (2-year mean)

Crop	Treatment	Land preparation	Residue management	Sowing/transplanting	Fertilizer management	Irrigation	Inter-culture	Chemical weed management	Plant protection	Harvest/threshing	Total input energy
		(MJ ha^−1^)									
Wheat	Residue management										
	Residue removal	818	–	1727	12,764	6955	–	322	13	450	23,050
	Residue retention	818	31,284	1727	12,764	6955	–	322	13	450	54,334
	TCE practice										
	CTTPR-CT	3272	13,772	1951	12,764	6955	–	34	13	450	39,211
	NPTPR-ZT	–	15,487	1652	12,764	6955	–	418	13	450	37,741
	ZTTPR-ZT	–	15,762	1652	12,764	6955	–	418	13	450	38,016
	ZTDSR-ZT	–	17,547	1652	12,764	6955	–	418	13	450	39,800
Maize	Residue management										
	Residue removal	818	–	437	12,743	5769	–	466	281	344	20,859
	Residue retention	818	30,176	437	12,743	5769	–	466	281	438	51,129
	TCE practice										
	CTTPR-CT	3272	13,252	560	12,743	5769	–	178	281	391	36,446
	NPTPR-ZT	–	14,885	396	12,743	5769	–	562	281	391	35,028
	ZTTPR-ZT	–	16,140	396	12,743	5769	–	562	281	391	36,283
	ZTDSR-ZT	–	16,074	396	12,743	5769	–	562	281	391	36,217

### Energy utilization pattern in cropping system

Energy utilization pattern of rice–wheat and rice–maize cropping systems was comparable. Conservation tillage-based crop establishment treatments reduced the energy consumption through fuel, water, and machinery (Fig. 2). Irrespective of the cropping systems, energy input through crop residue was the primary source of input energy in both the cropping systems. The energy input through crop residue was in the order of ZTDSR-ZT > ZTTPR-ZT > NPTPR-ZT > CTTPR-CT (Fig. 2). Table 5 shows that operation-wise, energy requirement was highest for crop residue management (~ 50% of total energy), followed by irrigation and fertilizer management. The conservation tillage-based crop establishment practices reduced the energy consumption in land preparation and irrigation by 4536–6544 MJ ha^−1^ and 8314–9843 MJ ha^−1^in rice–wheat and rice–maize rotations. Conservation tillage treatments increased the energy consumption through crop residue by 9–21% and 9–19% compared with CTTPR-CT treatment in rice–wheat and rice–maize system, respectively.

**Table 5:** Operation-wise energy use (MJ ha^−1^) in rice–wheat and rice–maize cropping systems as influenced by residue management and TCE practices (2-year mean)

	Treatment	Land preparation	Sowing/transplanting	Residue management	Fertilizer management	Irrigation	Inter-culture	Chemical weed management	Plant protection	Harvest/threshing	Total input energy
		(MJ ha^−1^)									
Rice–wheat	Residue management										
	Residue removal	2138	2379	–	28,529	29,678	35	565	82	900	64,307
	Residue retention	2138	2379	67,040	28,529	29,678	71	565	82	900	131,382
	TCE practice										
	CTTPR-CT	6544	2469	30,321	28,529	36,678	–	189	82	900	105,711
	NPTPR-ZT	2008	2170	32,959	28,529	26,835	–	573	82	900	94,057
	ZTTPR-ZT	–	2170	34,197	28,529	26,835	–	749	82	900	93,462
	ZTDSR-ZT	–	2708	36,602	28,529	28,364	212	749	82	900	98,146
Rice–maize	Residue management										
	Residue removal	2138	1090	–	28,508	28,492	35	709	349	794	62,115
	Residue retention	2138	1090	71,452	28,508	28,492	71	709	349	889	133,697
	TCE practice										
	CTTPR-CT	6544	1078	32,084	28,508	35,492	–	333	349	842	105,229
	NPTPR-ZT	2008	914	34,851	28,508	25,649	–	717	349	842	93,838
	ZTTPR-ZT	–	914	37,780	28,508	25,649	–	893	349	842	94,934
	ZTDSR-ZT	–	1452	38,190	28,508	27,178	212	893	349	842	97,624

### Output energy and energy productivity

During the fourth and fifth years of crop rotation, the impact of residue retention and tillage cum crop establishment practices was prominent on grain and straw yields of all the component crops (Supplementary Table 1). Crop residue retention increase in grain and straw yields that resulted in an increase in total output energy by 12,174, 15,596, and 23,972 MJ ha^−1^ in rice, wheat, and maize crop, respectively. Conservation tillage treatments (NPTPR-ZT, ZTTPR-ZT, and ZTDSR-ZT) increased the grain yield of rice, wheat, and maize by 1–15%, 14–23%, and 5–12% compared with CTTPR-CT, respectively (data not presented). Subsequently, the mean total output energy in conservation tillage treatments was highest in ZTDSR-ZT for all the component crops. The net energy output was higher from the maize and wheat crop, which were notably higher over the rice crop. In parallel, the energy ratio and energy productivity values were highest in maize and least in rice (Table 6).

**Table 6:** Crop productivity, input–output energy, and energy indices of rice, wheat, and maize crops as influenced by different cropping system, residue management, and TCE practices (2-year mean)

Crop	Treatment	Input energy (MJ ha^−1^)	Output energy (MJ ha^−1^)	Net energy return (MJ ha^−1^)	Energy ratio	Energy productivity (kg MJ^−1^)
Rice	Cropping system					
	Rice–wheat	59,152	164,784a	105,632a	3.06a	0.088a
	Rice–maize	61,913	163,272b	101,359a	2.98b	0.085b
	Residue management					
	Residue removal	41,257	157,941b	116,684a	3.90a	0.111a
	Residue retention	79,808	170,115a	90,307b	2.14b	0.062b
	TCE practice					
	CTTPR-CT	67,641	150,973d	83,331c	2.38c	0.069b
	NPTPR-ZT	57,563	159,411c	101,848b	3.06b	0.086b
	ZTTPR-ZT	57,049	168,876b	111,827a	3.33a	0.095a
	ZTDSR-ZT	59,876	176,852a	116,976a	3.31a	0.095a
Wheat	Residue management					
	Residue removal	23,050	178,671b	155,621a	7.80a	0.221a
	Residue retention	54,334	194,267a	139,932b	3.57b	0.101b
	TCE practice					
	CTTPR-CT	39,211	168,499c	129,288b	4.83b	0.133c
	NPTPR-ZT	37,741	186,494b	148,753a	5.86ab	0.166b
	ZTTPR-ZT	38,016	193,240ab	155,224a	6.04a	0.172a
	ZTDSR-ZT	39,800	197,642a	157,842a	6.01a	0.174a
Maize	Residue management					
	Residue removal	20,859	218,385b	197,526a	10.53a	0.332a
	Residue retention	51,129	242,357a	191,229a	4.74b	0.146b
	TCE practice					
	CTTPR-CT	36,446	214,675c	178,228c	6.69c	0.210c
	NPTPR-ZT	35,028	225,895b	190,868b	7.72b	0.241b
	ZTTPR-ZT	36,283	238,004a	201,721a	7.97a	0.252a
	ZTDSR-ZT	36,217	242,910a	206,693a	8.15a	0.253a

*a–d* different letters in continuous column are significantly different at *p* ≤ 0.05

Rice–maize rotation had a higher net energy return over rice–wheat (Table 7). Crop residue retention reduced the net energy return. The maximum increase in net energy return, energy ratio, and energy productivity was observed in ZTDSR-ZT treatment, which was 26, 32, and 32% higher for rice, wheat, and maize crops, respectively, when compared with CTTPR-CT (conventional practice).

**Table 7:** System productivity, output energy, and input–output energy relationship influenced by cropping system, residue management, and TCE practices (2-year mean)

Treatment	Input energy (MJ ha^−1^)	Output energy (MJ ha^−1^)	Net energy return (MJ ha^−1^)	Energy ratio	Energy productivity (kg MJ^−1^)
Cropping system					
Rice–wheat	97,844	351,253b	253,409b	4.04b	0.115b
Rice–maize	97,906	393,643a	295,737a	4.61a	0.139a
Residue management					
Residue removal	63,211	356,469b	293,258a	5.72a	0.168a
Residue retention	132,539	388,427a	255,888b	2.93b	0.086b
TCE practice					
CTTPR-CT	105,470	342,559d	237,089c	3.53c	0.104c
NPTPR-ZT	93,947	365,606c	271,658b	4.41b	0.128b
ZTTPR-ZT	94,198	384,498b	290,300a	4.70a	0.139a
ZTDSR-ZT	97,885	397,129a	299,243a	4.67a	0.137a

*a–d* different letters in continuous column are significantly different at *p* ≤ 0.05

### Energy-economics relations and multivariate analysis

Table 8 shows that energy indices and economic parameters had significant correlations. Total cost of cultivation (TCC) had significant negative association with output energy, net energy, energy ratio, and energy productivity parameters (*p* < 0.05). In contrast, input energy had non-significant correlation with total cost of cultivation, gross return, net return, and benefit–cost ratio. Net energy return and net economic return had a strong positive correlation (*p* < 0.001).

**Table 8:** Correlation coefficient (*r*) matrix of different energy and economic parameters (*n* = 48)

Parameter	IE	OE	NE	ER	EP	GR	TCC	NR	BCR
IE									
OE	0.40**								
NE	− 0.53***	0.56***							
ER	− 0.95***	− 0.11	0.75***						
EP	− 0.92***	− 0.06	0.77***	0.99***					
GR	− 0.10	0.56***	0.61***	0.26*	0.23				
TCC	0.18	− 0.48***	− 0.60***	− 0.35*	− 0.34*	− 0.74***			
NR	− 0.13	0.57***	0.64***	0.31*	0.29*	0.97***	− 0.89***		
BCR	− 0.14	0.55***	0.63***	0.32*	0.30*	0.95***	− 0.91***	1.00***	

*IE* input energy, *OE* output energy, *NE* net energy, *ER* energy ratio, *EP* energy productivity, *GR* gross return, *TCC* total cost of cultivation, *NR* net return, *BCR* B/C ratio

*Significant at *p* < 0.05

**Significant at *p* < 0.01

***Significant at *p* < 0.001

**Fig. 3:**
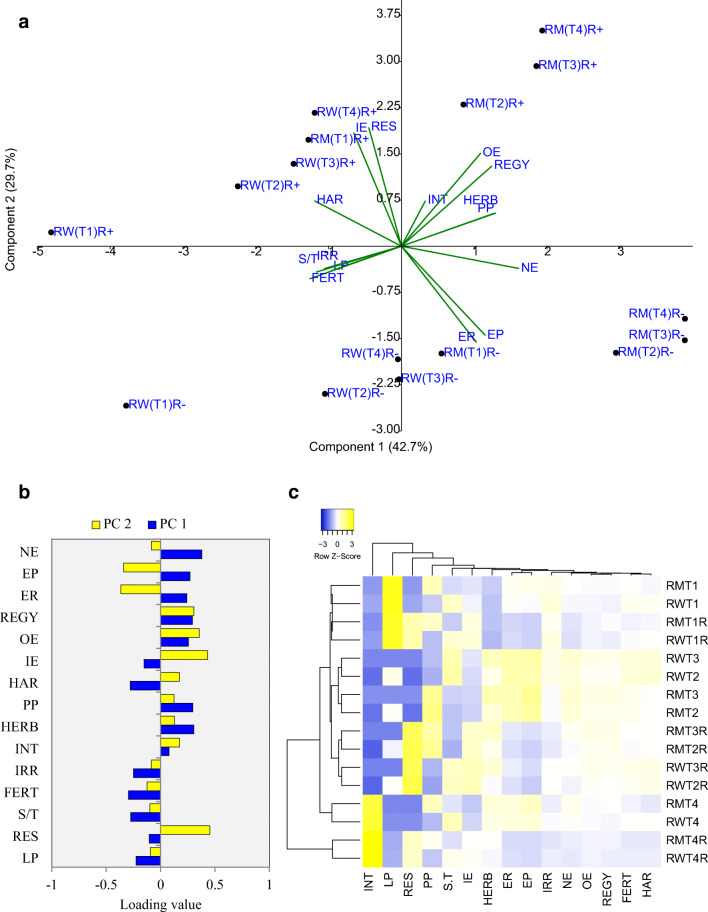
Scatter plot of treatments on PCA coordinates and their association with energy parameters (**a**). Loading value (correlation) of different variables for PC1 and PC2 (**b**). Heat map and cluster presentation of treatments based on the energy parameters (**c**). *LP* energy input for land preparation, *RES* crop residue energy input, *S/T* energy input for sowing/transplanting, *FERT* energy input for fertilizers application, *IRR* energy input for irrigation management, *INT* energy input for intercultural operation, *HERB* energy input for herbicide and its application, *PP* energy input for plant protection, *HAR* energy input for harvesting, *IE* input energy, *OE* output energy, *RGEY* system rice equivalent yield, *ER* energy ratio, *EP* energy productivity, *NE* net energy, *RW* rice–wheat, *RM* rice–maize, *T1* CTTPR-CT, *T2* NPTPR-ZT, *T3* ZTTPR-ZT, *T4* ZTDSR-ZT, *R−* residue removal, *R+* residue retention (for heat map, R stands for residue retention)

Scatter plot of treatments on PCA coordinates showed that residue retention and residue removal treatments are distinctly located on PCA coordinates (Fig. 3). Conservation tillage treatments ZTTPR-ZT and ZTDSR-ZT in rice–maize cropping system are positioned in right-hand-side coordinates with higher weightage of component 1 (42.7%). A close association between energy parameters like output energy, net energy, energy ratio, and energy productivity is also apparent from PCA graph (Fig. 3). Heatmap and cluster analysis also established the same, where treatments ZTTPR-ZT and ZTDSR-ZT were demarcated as the best treatments (clusters with close association) based on the energy parameters. The predicted regression models revealed that the association between total energy input and system productivity (systems rice equivalent grain yield) were either non significant (residue retention) or negative (residue removal) (Supplementary Fig. 1).

## Discussion

Conservation of non-renewable energy sources is a primary concern worldwide. Fossil fuels (e.g., diesel) are the directly non-renewable source of energy, where fertilizers are considered as indirect non-renewable energy source (Saad et al. 2016). After the USA, China, and Japan, India is the fourth largest consumer of oil and petroleum products (Kaplan 2009). The groundwater for irrigation (freshwater) is a directly renewable natural resource. These natural resources are limited and depleting fast. Hence, the efficient use of these resources through strategic changes in the agro-technique(s) is warranted to remain sustainable in the long run (Kumar et al. 2018; Venkatesh et al. 2019). The groundwater for irrigation is declining rapidly. Tube well is the primary source of irrigation in the IGP region and a remarkable fall in the groundwater table in rice–wheat growing regions has been observed in the last two to three decades (Gupta et al. 2002; Yadav et al. 2018), which warrants serious attention. As a result, the energy requirement for pumping of groundwater has increased by many folds, particularly in northwestern India—a rice–wheat dominating agro-region. Given the context, the relevance of CA practices in rice-based cropping systems for conservation of energy and natural resources would be a win–win situation.

### Tillage-based crop establishment practices and energy relation

Complete elimination of tillage in rice–wheat and rice–maize cropping systems (i.e., ZTTPR-ZT and ZTDSR-ZT) or only dry-tillage during rice season (NPTPR-ZT) could curtail the requirement of non-renewable energy source, i.e., diesel. Thus, the reduced use of fossil fuel in conservation tillage treatments is likely to reduce the load of greenhouse gases in the atmosphere and thus adds to ecosystem services (Busari et al. 2015; Gupta et al. 2016a). On the other hand, complete elimination of tillage or reduced tillage limits the oxidation of soil organic matter and thus has an advantage of lower emission of carbon dioxide (Dossou-Yovo et al. 2016; Ladha et al. 2016). Our results on soil parameters (data not presented) also support the fact that conservation tillage enhances C-sequestration and reduces soil carbon loss, being higher in ZTDSR-ZT and ZTTPR-ZT treatments (Nandan et al. 2019). Our results further demonstrate that the increased potential of energy conservation through conservation tillage practices in rice crop compared with that of wheat or maize crop is primarily because of higher energy use in tillage, transplanting, and irrigation in rice crop.

The advantage of conservation tillage on energy conservation is also attributed to the reduced requirement of irrigation as compared with conventional CTTPR-CT (Table 1). Constructive changes in soil attributes and a different rice growing ecology under conservation tillage practices substantially reduced the irrigation requirement in rice crop (Nandan et al. 2019; Gathala et al. 2019). For instance, in conventional puddled condition, standing water is maintained throughout the rice growing season, where in conservation tillage cum crop establishment practices flooded condition was avoided, thereby reducing energy investment in irrigation to rice. Conservation tillage treatments improved the soil environment (particularly soil aggregation and SOC) that might have helped to curtail (28–33%) the water requirements as compared with CTTPR-CT (Nandan et al. 2018a; Nandan et al. 2019). The higher use of herbicides in conservation tillage treatments had marginal influence on total energy input value as these herbicides were applied in small quantities.

The yield advantage with conservation tillage practices (particularly ZTTPR-ZT and ZTDSR-ZT) over CTTPR-CT treatment directly reflected in the higher energy output, which is primarily because of improvement in soil quality parameters and favorable crop growing environment under conservation tillage treatments. The mid-term or long-term impact of conservation tillage practices on soil quality and crop productivity has been observed in most of the earlier studies (Jat et al. 2013; Ladha et al. 2016). As the positive impact of conservation tillage on soil properties and crop productivity is likely to increase over time, an increase in energy output is therefore expected with long-run adoption of conservation tillage in rice-based rotations.

Fertilizer energy input accounted for a major share in the total input energy. Changes in soil moisture and tillage regimes in conservation tillage practices may influence the crop response to fertilizer application—a key yield determining factor. Particular to tropical rice soils, conventional submerged soil conditions have an advantage for nutrient mobilization (particularly N, P, and Zn); on the contrary, it also allows losses of some nutrients from soil profile, and thus crops with conservation tillage practices may have a differential fertilizer requirement that must be looked into.

### Crop residue retention and energy relations

Crop residue retention is an integral component of CA and it strongly influences the energy inflow. Indeed, in some of the previous studies, crop residue has not been taken as a component for estimation of energy budgeting as the retained or incorporated crop residues is an integral part of the soil system (Saad et al. 2016; Ronga et al. 2019). Indeed, in a country like India and in other south Asian countries, crop residues are widely used for cattle feeding, thatching of houses, and domestic fuel (Devi et al. 2017), whereas in the large parts of the IGP, crop residues of rice and wheat crop are burnt as an easy disposal of the left-out residues after combine harvesting (Lohan et al. 2018; Ravindra et al. 2019). However, this practice has a notable adverse impact on the environment (Kumar et al. 2015; Gupta et al. 2016b; Singh et al. 2019). Burning of residues in the IGP has drawn the attention of researchers and planners as this practice has several adverse impacts on productivity of soil and environment. The benefits of residue retention in tropical agro-regions are significant and improve crop productivity and soil health (Mandal et al. 2007; Venkatesh et al. 2013). Our study suggested that the yield benefits from residue retention could not compensate the energy input through residue retention. Nevertheless, cereal–cereal rotations like rice–wheat and rice–maize produce a large amount of biomass, and recycling of one third of total straw biomass—as a renewable source of bio-energy—is therefore a sustainable approach and also a socially adaptable approach in a country like India. The total available crop residue in the IGP region is ~ 42 million tons that have a fertilizer replacement value of about 3.6 billion Indian national rupees year^−1^. Hence, the bio-energy inflow in CA practices must be looked in a different perspective.

### Cropping system and energy relations

Cropping system and associated management practices directly influence the energy use and energy productivity (Tuti et al. 2012). Our results demonstrate that both rice–maize and rice–wheat are comparable for their energy requirement; however, the higher productivity potential of rice–maize rotation resulted in higher energy productivity and is thus recommended. Conservation tillage treatments improve the productivity of all the crops being higher in wheat (14–23%) followed by rice (9–15%). Nevertheless, the impact of conservation agriculture on energy productivity and energy ratio was more prominent on rice–wheat rotation over rice–maize rotation (Supplementary Fig. 2). Therefore, under conservation agriculture, rice–wheat would be the strategic choice over rice–maize system, particularly in the IGP region.

### Energy-economics relationship

Our results support the “conservative hypothesis” of energy-economic relations. In the present study, input energy did not influence the gross return and net return. In fact, both crop productivity and economic return were higher in conservation tillage or CA practices, where energy requirement was substantially reduced. Hence, the CA practices could be a potential alternative for elevating the energy productivity in rice-based cropping systems of the IGP, where the economic status of the farmers is also not much favorable.

## Conclusions

The study therefore advocates CA practices in rice–wheat/maize cropping systems to curtail the energy inputs, conserving natural resources, and sustaining the crop productivity. Our results recommended that CA could be a potential alternative to tillage and input-intensive conventional rice-based production system (CTTPR-CT), which are practiced in large scale in South Asia. Apart from the benefits of soil health restoration and production sustainability, adoption of CA in rice-based production systems adequately minimized the energy investment through non-renewable fossil fuels (land preparation, irrigation), and therefore adds to the ecosystem services and cleaner production. In the context of degrading natural resources (particularly groundwater and soil quality) in the IGP region, conservation agriculture in rice-based cropping systems could be the strategic option. Conservation tillage treatments particularly ZTTPR-ZT and ZTDSR-ZT could upscale the energy productivity and conserve the non-renewable energy resources. In tropical regions, retention of a part of crop residue in high biomass production systems is a sustainable approach as it has the notable positive impact on soil health associated with yield benefits. The study also proposed that higher energy inputs are not essentially the primary driver of profitable production system. Thus, minimal use of energy sources through CA practices is likely to have a large impact on production economies and environments.

## Electronic supplementary material

ESM 1 (DOCX 70 kb)
