# Supplementary material for: Potential of conservation agriculture modules for energy conservation and sustainability of rice-based production systems of Indo-Gangetic Plain region
Source: Environ Sci Pollut Res Int. 2020 Aug 18;28(1):246–61. doi: 10.1007/s11356-020-10395-x (PMC7782432; doi:10.1007/s11356-020-10395-x)
Supplement: Supplementary file 1 — (DOCX 70 kb) [file 11356_2020_10395_MOESM1_ESM.docx]

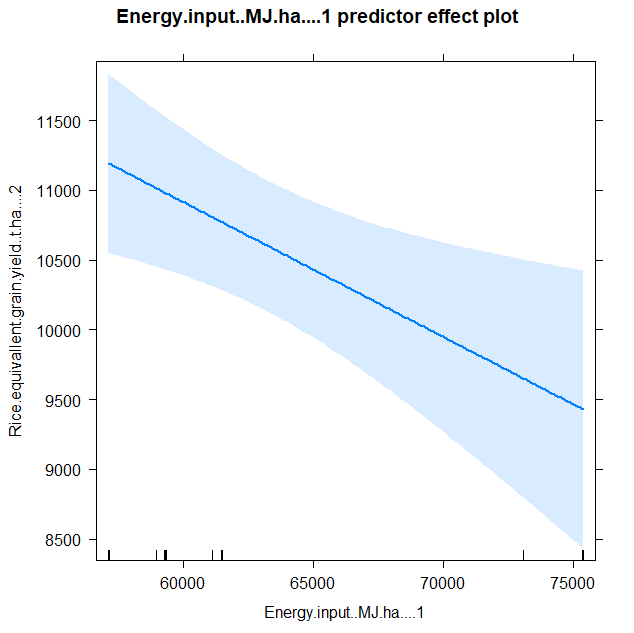


Residue removal (*n* = 24)

REGY = - 0.096TIE + 16702
R² = 0.280 ; *p* = 0.0163

Rice equivalent grain yield (kg ha^-1^)


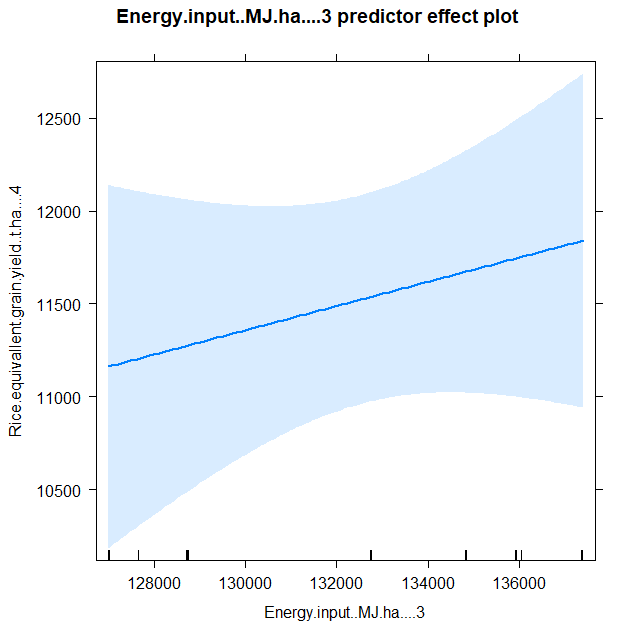


Residue retention (*n* = 24)

REGY = = 0.065TIE + 2865
R² = 0.040; *p* = 0.336 (*ns*)

Total input energy (MJ ha^-1^)

**Supplementary Fig. 1** Predicted regression model of system productivity (rice equivalent grain yield) and total input energy.

**Supplementary Fig. 2** Total input and output energy, net energy, energy ratio, and energy productivity subjected to different treatments of tillage cum crop establishment practices and residue management in rice-wheat and rice-maize rotations. The values are mean of two years (2013-2014 and 2014-2015). The error bar represents standard error of mean.

**Supplementary Table 1** Grain, straw/stover and system productivity as by cropping system, residue management and TCE practices (2-year mean)

|  | Rice |  | Wheat |  | Maize |  | System productivity# | |
| --- | --- | --- | --- | --- | --- | --- | --- | --- |
| Treatment | Grain yield  (t ha^-1^) | Straw yield  (t ha^-1^) | Grain yield  (t ha^-1^) | Straw yield  (t ha^-1^) | Grain yield  (t ha^-1^) | Straw yield  (t ha^-1^) | Grain yield  (t ha^-1^) | Straw yield  (t ha^-1^) |
| Cropping system |  |  |  |  |  |  |  |  |
| Rice-wheat | 4.74a | 7.24a | 5.27 | 8.30 | - | - | 10.01b | 15.53b |
| Rice-maize | 4.66a | 7.20a | - | - | 7.18 | 9.42 | 11.84a | 16.62a |
|  |  |  |  |  |  |  |  |  |
| Residue management |  |  |  |  |  |  |  |  |
| Residue removal | 4.49b | 6.99b | 5.07b | 7.93b | 6.88b | 8.83b | 10.47b | 15.37b |
| Residue retention | 4.90a | 7.45a | 5.47a | 8.67a | 7.47a | 10.01a | 11.38a | 16.79a |
|  |  |  |  |  |  |  |  |  |
| TCE practice |  |  |  |  |  |  |  |  |
| CTTPR-CT | 4.42c | 6.52c | 4.63b | 7.66c | 6.71c | 8.74c | 10.09d | 14.73d |
| NPTPR-ZT | 4.48c | 7.13b | 5.29a | 8.27b | 7.04b | 9.23b | 10.65c | 15.88c |
| ZTTPR-ZT | 4.83b | 7.44ab | 5.48a | 8.57ab | 7.44a | 9.70a | 11.30b | 16.57b |
| ZTDSR-ZT | 5.06a | 7.79a | 5.68a | 8.68a | 7.50a | 10.01a | 11.65a | 17.14a |

*a-d* different letters in continuous column are significantly different at *p* ≤ 0.05

# sum of component crops yields in rice-wheat/rice-maize rotation
